# Cytotoxicity of apo bovine α-lactalbumin complexed with La^3+^ on cancer cells supported by its high resolution crystal structure

**DOI:** 10.1038/s41598-018-38024-1

**Published:** 2019-02-11

**Authors:** Deepthi S. Yarramala, Prem Prakash, Dnyanesh S. Ranade, Sejal Doshi, Prasad P. Kulkarni, Prasenjit Bhaumik, Chebrolu Pulla Rao

**Affiliations:** 1 Department of Chemistry, Indian Institute of Technology Bombay, Powai, Mumbai 400 076 India; 2 Department of Biosciences and Bioengineering, Indian Institute of Technology Bombay, Powai, Mumbai 400 076 India; 3 Bioprospecting Group, Agharkar Research Institute, Pune, 411 004 India; 4 Department of Metallurgical Engineering and Materials Science, Indian Institute of Technology Bombay, Powai, Mumbai 400 076 India

## Abstract

Cancer remains one of the biggest threats to human society. There are massive demands for compounds to selectively kill cancerous cells. Earlier studies have shown that bovine α -lactalbumin made lethal to tumor cells (BAMLET) becomes cytotoxic against cancer cells in complex with oleic acid {Hoque, M. *et. al*., *PLoSOne* **8**, e68390 (2013)}. In our study, we obtained bovine α-lactalbumin complexed with lanthanum ion (La^3+^-B-α-LA) and determined its high resolution crystal structure. The natural calcium binding site of bovine α-lactalbumin is replaced by lanthanum. The La^3+^ complex formation by B-α-apo-LA was also supported by various biophysical methods. Interestingly, our complex, La^3+^-B-α-LA exhibits much greater anticancer activity against breast cancer cells as compared to the reported BAMLET-oleic acid complex. This study shows that La^3+^-B-α-LA complex is preferentially more toxic to MCF-7 cells as compared to KB (oral cancer) and HeLa (cervical) cells, while almost non-toxic to the healthy cells that we studied. Our data indicates that the cytotoxicity of La^3+^-B-α-LA against cancer cells is through apoptotic path way. The higher anticancer activity of La^3+^-B-α-LA is attributable to the requisite structural changes induced in the protein by La^3+^ binding as supported by the crystal structure of the complex.

## Introduction

Development of new methods for cancer treatment and prevention are always in high demand^1^. Human α-lactalbumin (H-α-LA) made lethal to tumor cells (HAMLET) and bovine α-lactalbumin (B-α-LA) made lethal to tumor cells (BAMLET), both cause death to the cancer cells through apoptosis but sparing the healthy cells^2–7^. Conversion of native α-lactalbumin into the apoptosis inducing form, BAMLET/HAMLET, involves partial unfolding of the protein followed by complexing with oleic acid followed by stabilising the partially folded conformation^8–17^. The detailed mechanism by which HAMLET/BAMLET acts as anti-cancer agent is under explored^18^. Some recent studies report the binding of B-α-LA to nanoclusters^19,20^ and nanoparticles^21^ of gold. Such nano-species show alternative pathways for stabilizing the unfolded state of B-α-LA so that the resulting protein-metal nano-composites express their required properties^22^. Though the BAMLET like structure could be synthesized from B-α-LA/ H-α-LA by different methods^11,12,19,23^, to our knowledge no such method uses any metal ion, further no crystal structure of BAMLET/HAMLET is known. The formation of protein-inorganic hybrids is greatly influenced by the exposed residues, binding core, coordination characteristics of the inorganic ion and the medium. The protein-inorganic hybrids show wide range of applications in catalysis, as drug carriers and in drug delivery, and in killing cancer cells^24–26^.

In the present study, we focused on the metallation of apo-B-α-LA by La^3+^ in order to complex as well to induce structural changes into the protein. The metallation was studied by spectroscopy and calorimetry. The coordination, protein conformation and structural aspects of La^3+^ bound B-α-LA were established by analyzing the crystal structure. The selective anti-cancer activity of this complex was studied using different types of cancer cell lines. The details of apoptosis were probed in one case, and the activity was compared accordingly with that reported in the literature.

## Results and Discussion

### Binding of La^3+^ to apo-B-α-LA

**Figure 1:**
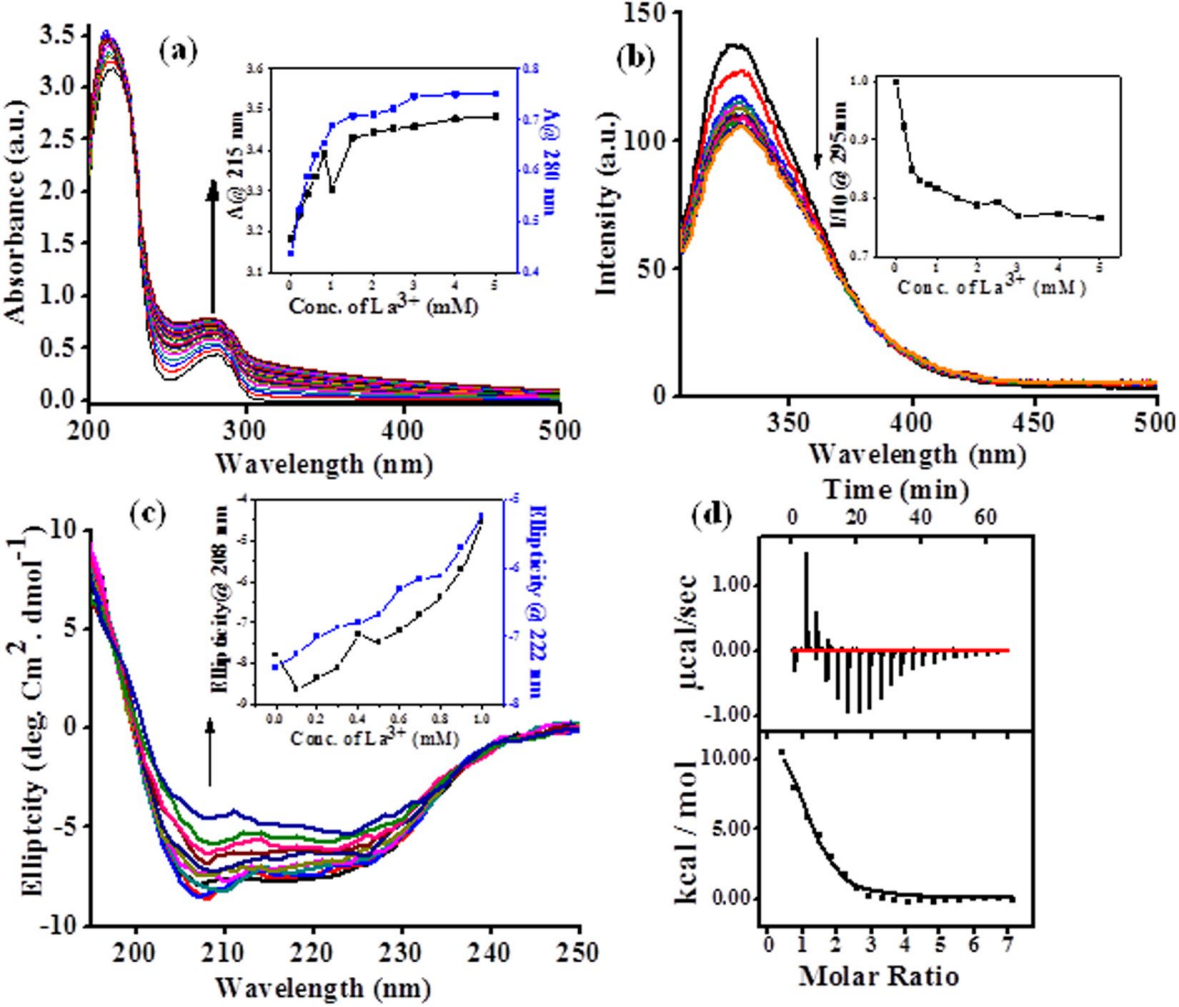
Spectral data of apo-bovine-α-LA titrated with different La^3+^ as per the details given in the section on “Methods”: (**a**) Absorption spectra obtained during the titration. The inset is the absorbance *vs*. concentration plots at 215 and 280 nm. (**b**) Fluorescence emission spectra (λ_ex_ = 295 nm). The inset is the I/I_0_ *vs*. concentration plot. (**c**) Circular dichroism spectra. The inset is the ellipticity *vs*. concentration plots at 222 and 208 nm. (**d**) Calorimetric titration isotherm. The concentration of La^3+^ used in each of these experiment can be seen from the x-axis of the inset of the corresponding figure.

The interaction followed by binding of La^3+^ with apo-B-α-LA was studied by analytical methods, thermodynamics and spectroscopy. The ICP-AES data (see Supplementary Data, S01) of the isolated complex carried out as per the details given in the section on Methods fits well with one La^3+^ ion per protein. The spectra exhibited an increase in the absorbance of both the 280 and 215 nm bands as the concentration of the added La^3+^ increases (Fig. 1a). While the 280 nm band suggests the influence of binding on aromatic amino acid residues, the 215 nm band reflects the interaction of La^3+^ ion with the peptide bond as well as the free carboxylate groups. The fluorescence emission spectra were recorded upon addition of La^3+^ to apo-B-α-LA at λ_ex_ = 295 nm and only a maximum of ~20% quenching was observed (Fig. 1b), supporting that the La^3+^ binding to protein does not substantially alter the structure where the aromatic side chains are present. The CD spectra exhibited marginal ellipticity changes in 222 and 208 nm bands (Fig. 1c) and thus supports minimum conformational changes in the secondary structure of the protein. If an ion binds in the pocket meant for Ca^2+^ binding, such binding is expected to bring only minimal spectral changes.

The ITC titration exhibited clear cut isotherm supporting the binding of La^3+^ to the protein (Fig. 1d) and the data fits well to one La^3+^ ion per protein molecule with a binding constant (K_a_) of (7.3 ± 1.8) × 10^4^ M^−1^. The enthalphy and entropy components of the La^3+^ binding are (1.25 ± 0.1) × 10^4^ cal/mol and 64.4 cal/mol/deg respectively as derived from the ITC data. The La^3+^ binding pocket was identified based on the crystal structure established as given in this paper.

### Structural proof for the binding of La^3+^ in the Ca^2+^ binding site of apo-B-α-LA

The La^3+^ complex of apo-B-α-LA was crystallized as per the details given in the experimental section and the structure of the complex was established by single crystal X-ray diffraction at a resolution of 1.85 Å with one molecule in the asymmetric unit in *P*3_1_21 space group (Table 1). The binding of La^3+^ in the Ca^2+^ pocket was confirmed. Further, we have also addressed the conformational changes noticed in the protein backbone upon forming the complex.

**Table 1:** Data collection and refinement statistics of La^3+^-B-α-LA.

**A**. ***Data collection statistics***	
Wavelength (Å)	1.5418
Temperature (K)	100
Space group	*P*3_1_21
Unit cell constants *a*, *b*, *c* (Å) *α*, *β*, *γ* (°)	*a* = *b* = 47.45, *c* = 89.84 *α* = *γ* = 90, *β* = 120
Resolution (Å)	30.0–1.85 (1.95–1.85)
Measured reflections	115075 (9245)
Unique reflections	10499 (1496)
Mean *I*/*σ*(*Ι*)	25.6 (3.6)
Completeness (%)	100.0 (99.9)
*R*_merge_ (%)	6.3 (48.4)
*R*_meas_ (%)	6.6 (52.9)
Redundancy	11.0 (6.2)
No. of molecules/asymmetric unit	1
CC_1/2_ (%)	100.0 (80.7)
Wilson B factor (Å^2^)	30.4
**B**. ***Refinement statistics***	
Resolution (Å)	20.0-1.85
Working set: number of reflections	9963
Test set: number of reflections	524
*R*_factor_ (%)	19.7
*R*_free_ (%)	21.6
Protein atoms	995
No. of Lanthanum ion	1
No. of sulphate ion	1
No. of glycerol molecule	1
No. of water molecules	62
r.m.s.d. (bond distance) (Å)	0.007
r.m.s.d. (bond angle) (°)	1.421
***Overall average B-factor (Å*** ^***2***^ ***)***	31.0
***Estimated coordinate error (*** **Å** ***)***	
Based on maximum likelihood	0.13
Based on *R*_free_	0.12
***Protein-geometry (PROCHECK)***	
Ramachandran plot allowed (%)	99.1
Ramachandran plot generously allowed (%)	0.0
Ramachandran plot outliers (%)	0.9
***PDB code***	6IP9

^a^Values in parentheses correspond to highest resolution shell.

${R}_{meas}={{\rm{\Sigma }}}_{hkl}\sqrt{\frac{n}{n-1}}{{\rm{\Sigma }}}_{j=1}^{n}|{I}_{hkl,j}-\langle {I}_{hkl}\rangle \,|/{{\rm{\Sigma }}}_{hkl}{{\rm{\Sigma }}}_{j}{I}_{hkl,j}$, where $\langle {I}_{hkl}\rangle$is the average of symmetry related observations of a unique reflection.

**Figure 2:**
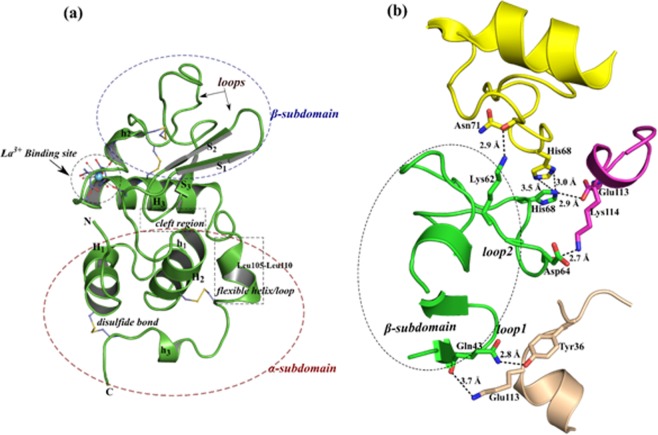
(**a**) The overall structure of La^3+^-B-α-LA represented as cartoon and the bound La^3+^ion is shown as a cyan sphere. The secondary structural elements are marked as H, α-helix; h, 3_10_ helix; S, β-strand and the disulphide bonds are shown in yellow. The residues involved in La^3+^ ion binding and disulphide bridge formation are shown as ball and stick. The polar interactions with bound La^3+^ ion are shown as dotted lines. (**b**) The contacts between the symmetry related molecules of La^3+^-B-α-LA complex in the lattice are shown. The secondary structural elements of the four molecules are shown in green, magenta, yellow and wheat colour. The residues are shown as sticks. The polar interactions are presented with distances in Å.

The numbering of the residues in La^3+^-B-α-LA structure is same as that given in the crystal structure of Ca^2+^-B-α-LA (PDB ID = 1F6S)^27^. The overall structural fold of the La^3+^-B-α-LA complex is almost identical to the structure of apo-B-α-LA^27^. The structural superposition of La^3+^-B-α-LA with that of Ca^2+^-B-α-LA produced a root mean square deviation (r.m.s.d.) value of 0.54 Å. The structure of the complex La^3+^-B-α-LA is composed of four α-helices and three antiparallel β-strands (Fig. 2a). A flexible loop (residues 105–110) below the cleft region adopts a helical conformation in La^3+^-B-α-LA complex (Fig. 2a). The structure can be divided into α and β sub-domains and the presence of a cleft region clearly demarcates the existence of these two sub-domains. The α−sub-domain (residues 1–34 and 86–122) is bigger than the β-sub-domain (residues 35–85). The smaller β-sub-domain comprises of antiparallel β-sheets, irregular loops and a 3_10_ helix (Fig. 2a). In addition, the two loop regions of the β-sub-domain composed of amino acid residues, i.e., Gln43-Ser47 (loop 1) and Lys62-Gln65 (loop 2), are exposed to the solvent in the Ca^2+^-B-α-LA structure. On the contrary, those two loop regions of the β-sub-domain are stabilized by three symmetry related molecules in the lattice of La^3+^-B-α-LA structure (Fig. 2b). The average thermal B-factors of the residues from the loop 1 and loop 2 of the La^3+^-B-α-LA structure are 19.1 and 15.1 Å^2^ respectively. Conversely, the average B-factor of the residues from loop 1 and loop 2 of Ca^2+^-B-α-LA complex are 73.6 and 66.7 Å^2^ respectively. The La^3+^-B-α-LA forms an interfacial contact with the symmetry related molecules at the edge of the two domains by forming hydrogen bonds (Fig. 2b). There are 62 water molecules in the crystal structure of La^3+^-B-α-LA with an average B-factor of ~34.9 Å^2^, but ranges from 8.1 to 48.6 Å^2^.

#### Coordination characteristics of La^3+^ bound to protein in the crystal structure

**Figure 3:**
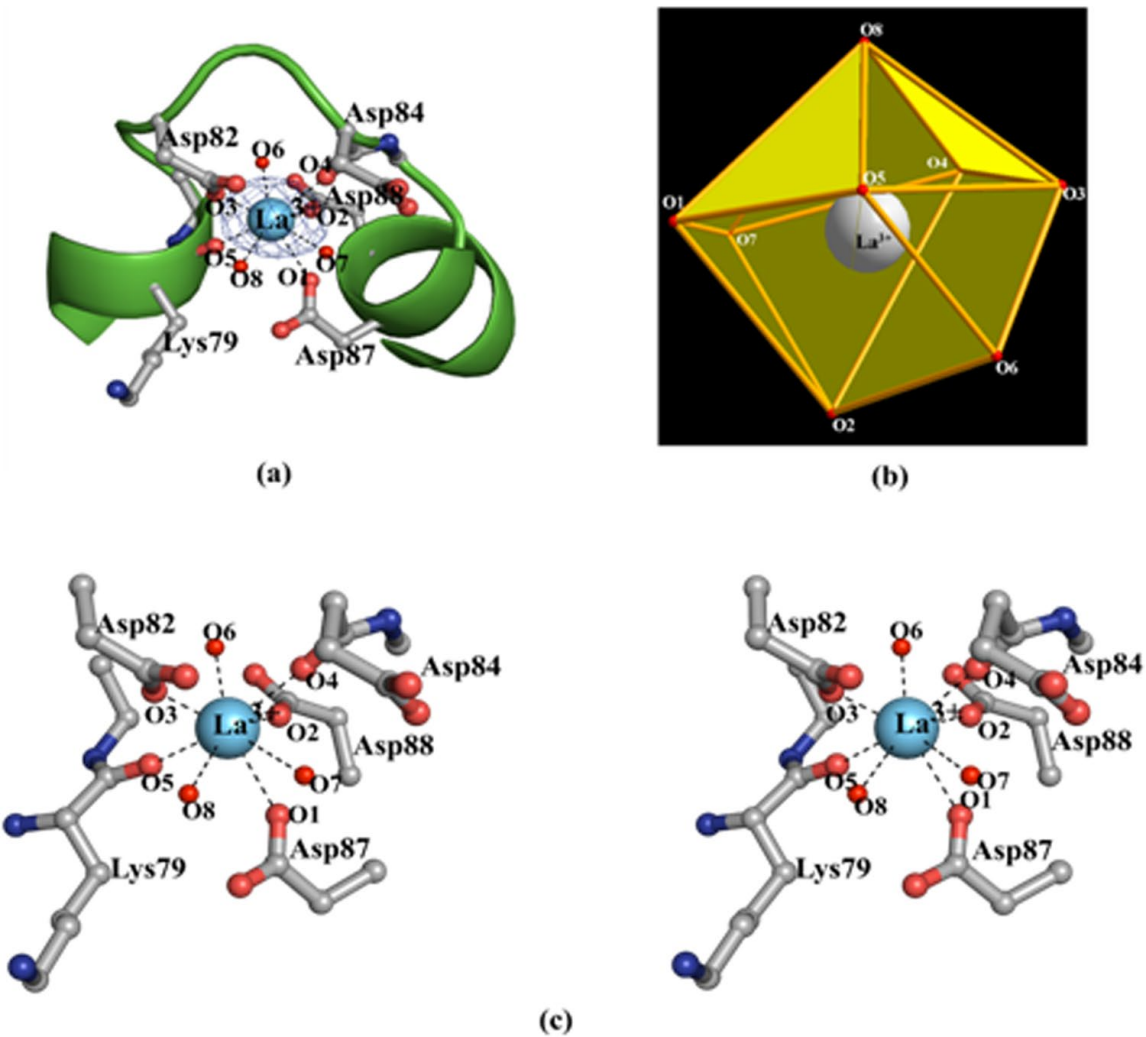
La^3+^ primary coordination as obtained from the crystal structure of La^3+^-B-α-LA complex: (**a**) Electron density map (*F*_*o*_*-F*_*c*_ map contoured at 4σ level) for La^3+^ in the calcium binding cleft. (**b**) Dodecahedral geometry of La^3+^ ion with eight coordination as observed in the La^3+^-B-α-LA crystal structure. (**c**) Zoomed in stereo view showing the interactions of La^3+^ ion with residues and the water molecules in case of La^3+^-B-α-LA structure. The protein residues are shown as ball and stick model. The water molecules are shown as small red spheres. The bound La^3+^ ion is shown as sky blue sphere.

The clear electron density defines the correct position of the La^3+^ ion in the Ca^2+^-binding cleft of holo-B-α-LA structure. The La^3+ ^ion is located in the same binding core as described previously for other metal ion bound structures of B-α-LA^28–30^. In the present structure, the La^3+^ is bound to the protein through the side chain carboxylate groups of Asp82, Asp87 and Asp88, and the main chain amide carbonyl groups of Lys79 and Asp84 (Fig. 3a,c) in addition to three water molecules (W1, W2 and W3). The corresponding La^3+^-O bond distances are 2.48, 2.63, 2.41, 2.1, 2.42, 2.7, 2.5, and 2.56 Å, and the bond angles range from 56.9 to 158.6° (see Supplementary Data, S02).

In the structure of La^3+^-B-α-LA, the La^3+^ shows eight coordination with dodecahedral geometry (Fig. 3b) while it is pentagonal bipyramidal w.r.t. Ca^2+^ in case of the Ca^2+^-B-α-LA structure. In the La^3+^-B-α-LA structure, the five coordinations, viz., Asp82, Asp87, Asp88 and two water molecules (W2 and W3) form a pentagon while the Lys79, Asp84 and W1 forms a triangle. In this triangle, the Asp 84 is on one side of the pentagon and the Lys79 and W1 are on the opposite side to this, while La^3+^ sits at the centre. The O···O bond distances in this pentagon range from 2.48 to 3.48 Å and O···O···O angles range from 95.6 to 113.5° supporting that the pentagon is highly puckered.

#### Comparison between the Ca^2+^ and La^3+^ coordination cores

**Figure 4:**
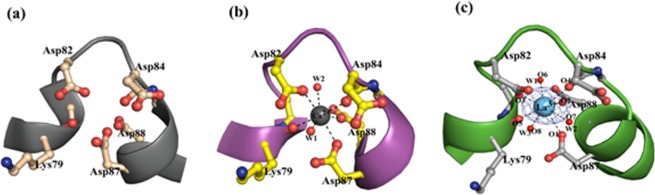
Comparison of the coordination core for the metal ion binding cleft in, (**a**) apo-B-α-LA^27^, (**b**) Ca^2+^-B-α-LA^27^ and (**c**) La^3+^-B-α-LA (present study) taken from the corresponding crystal structures.

The coordination cores of La^3+^ and Ca^2+^ bound to protein (B-α-LA) were compared. There are only two water molecules in the Ca^2+^-B-α-LA structure. The average La^3+^···O distance observed in the present structure (~2.5 Å) is longer than that observed for the average Ca^2+^···O distance (2.37 Å) by ~0.18 Å, supporting that the metal ion binding core expands in case of La^3+^ bound structure. The analysis revealed that La^3+^ is coordinated in the metal binding cleft of B-α-LA with an additional water molecule as compared to that in the Ca^2+^ bound structure. Additionally, some unique conformational changes are observed for the amino acid side chains involved upon binding to La^3+^ (Fig. 4c). The Asp82 and Asp87 side chains are in a similar plane for both the apo-B-α-LA and Ca^2+^-B-α-LA structures, while in La^3+^-B-α-LA, the side chain of Asp82 is rotated and hence the carboxylate group is almost perpendicular to what was observed in case of apo- (Fig. 4a) and Ca^2+^ structures (Fig. 4b).

#### Comparison of the La^3+^-B-α-LA structure with that of the apo-B-α-LA and Ca^2+^-B-α-LA

**Figure 5:**
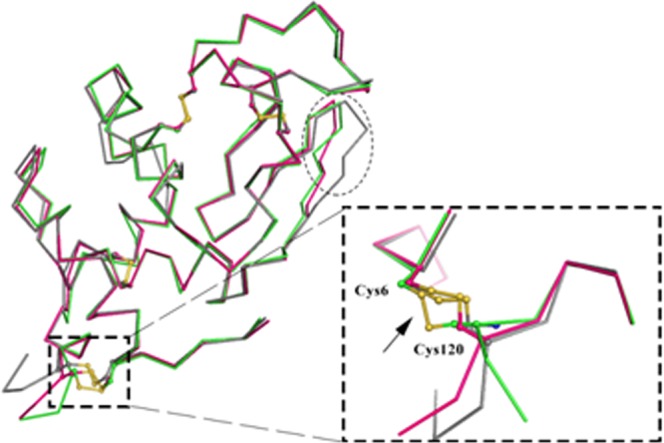
Superposition of the backbone structures of La^3+^-B-α-LA (green) with apo-B-α-LA (grey) and Ca^2+^-B-α-LA (magenta) showing major structural changes at disulphide linkage represented as dashed rectangle (zoomed view shown in inset), and the dotted circle represents a major change in loop region.

The structural comparison of La^3+^-B-α-LA with its apo- and holo-(Ca^2+^)-B-α-LA was performed and the analysis revealed some changes in the metal binding residues of the La^3+^-B-α-LA structure. Apart from these differences, there are some conformational changes in the protein backbone of La^3+^-B-α-LA as given in Fig. 5. The disulphide bond between Cys6-Cys120 in La^3+^-B-α-LA is flipped into an opposite orientation as compared to apo-B-α-LA and Ca^2+^-B-α-LA. The flexible loop region consisting of the residues Val42 to Ser47 exhibited conformational change as compared to two other B-α-LA structures reported^27^. The overall difference in r.m.s.d. values calculated after superposition of La^3+^-B-α-LA with apo-B-α-LA and Ca^2+^-B-α-LA resulted in values of 0.89 and 0.54 Å, respectively. The average B-factors of the main chain atoms are 29.0, 38.3 and 48.4 Å^2^ respectively for La^3+^-B-α-LA, apo-B-α-LA and Ca^2+^-B-α-LA. The average B factors suggest that the structure of the polypeptide in the La^3+^-B-α-LA complex is more rigid as compared to the other two structures. This implicates that the binding of La^3+^ ion and polar interfacial contacts among the symmetry related molecules stabilize the overall structure of B-α-LA when compared to the apo-form and or the Ca^2+^ bound form. When these structures were overlaid, several conformational changes were revealed in both the main chain as well in the side chains in case of La^3+^-B-α-LA structure when compared to its apo-form, as can be noticed from the encircled regions in Fig. 5.

### Biocompatibility of the La^3+^-B-α-LA complex

**Figure 6:**
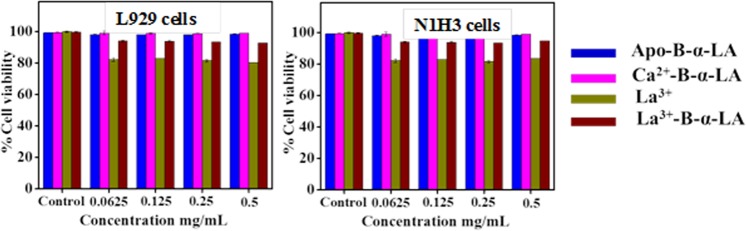
Bar diagram for the cell viability *vs*. different concentrations of samples treated with the healthy cells, viz., (**a**) L929 cells and (**b**) N1H3 cell lines.

The La^3+^-B-α-LA complex reported in this paper was examined for its biocompatibility with normal mouse fibroblast cells (L929) and N1H3 cells by performing the SRB assay. While the La^3+^ salt alone showed cell viability of 82–85%, the La^3+^-B-α-LA complex showed >95%. In order to check the effect of concentration, four different concentrations were used for the cell viability studies. In the concentration range of 4.5 to 35 µM studied, the La^3+^-B-α-LA complex showed cell viability of >90–95% (Fig. 6) suggesting the non-toxic nature of this complex and hence the studies were extended to evaluate the anticancer property of La^3+^-B-α-LA complex.

### Cytotoxicity of the La^3+^-B-α-LA complex

**Figure 7:**
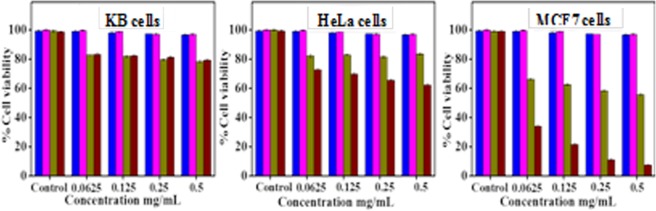
Bar diagram for the cell viability *vs*. the cells (KB cells, HeLa cell and MCF-7 cell lines) that get treated by the samples. The colour coding: apo-B-α-LA (blue), Ca^2+^-B-α-LA (magenta), La^3+^ (olive green) and La^3+^-B-α-LA (maroon).

The native form of the calcium bound protein (Ca^2+^-B-α-LA) does not exhibit any anti-cancer activity, however, exhibits enhanced effect when it undergoes conformational change to that of BAMLET that is stabilised by oleic acid as reported in the literature^11^. Therefore, herein, we explore the anticancer activity of La^3+^-B-α-LA on HeLa (cervical cancer cells), KB cells (oral cancer cells) and MCF-7 cells (breast cancer cells), since the binding of La^3+^ induces some conformational changes and stabilizes a structure that is different from that of apo- and or Ca^2+^ structure. It was observed that La^3+^-B-α-LA shows anti proliferation effect on KB, HeLa and MCF-7 cells (Fig. 7). In the concentration range 4–35 µM, the La^3+^-B-α-LA brings cell death of ~15–20% in case of KB cells and ~30–35% in case of HeLa cell lines. The anti-cancer activity of La^3+^-B-α-LA complex on MCF-7 cell lines showed ~70–93% of anticancer effect, whereas the simple La^3+^ salt and the apo-protein showed only 35–40% and 1–2% respectively, suggesting that the anti-cancer effect results primarily from the conformational/structural changes induced in the protein by La^3+^ binding, since the contribution from the bare La^3+^ is less than half of that exhibited by the La^3+^-B-α-LA complex. Thus, the cell culture studies reveal that La^3+^-B-α-LA complex at a concentration of 0.35 µM specifically kills the breast cancer cells sparing the healthy ones and its anticancer property follows a trend, i.e., MCF-7 (~93%)≫ > HeLa (~40%) > KB (~20%).

#### Fluorescence microscopy studies of the La^3+^-B-α-LA complex in MCF-7 cells

**Figure 8:**
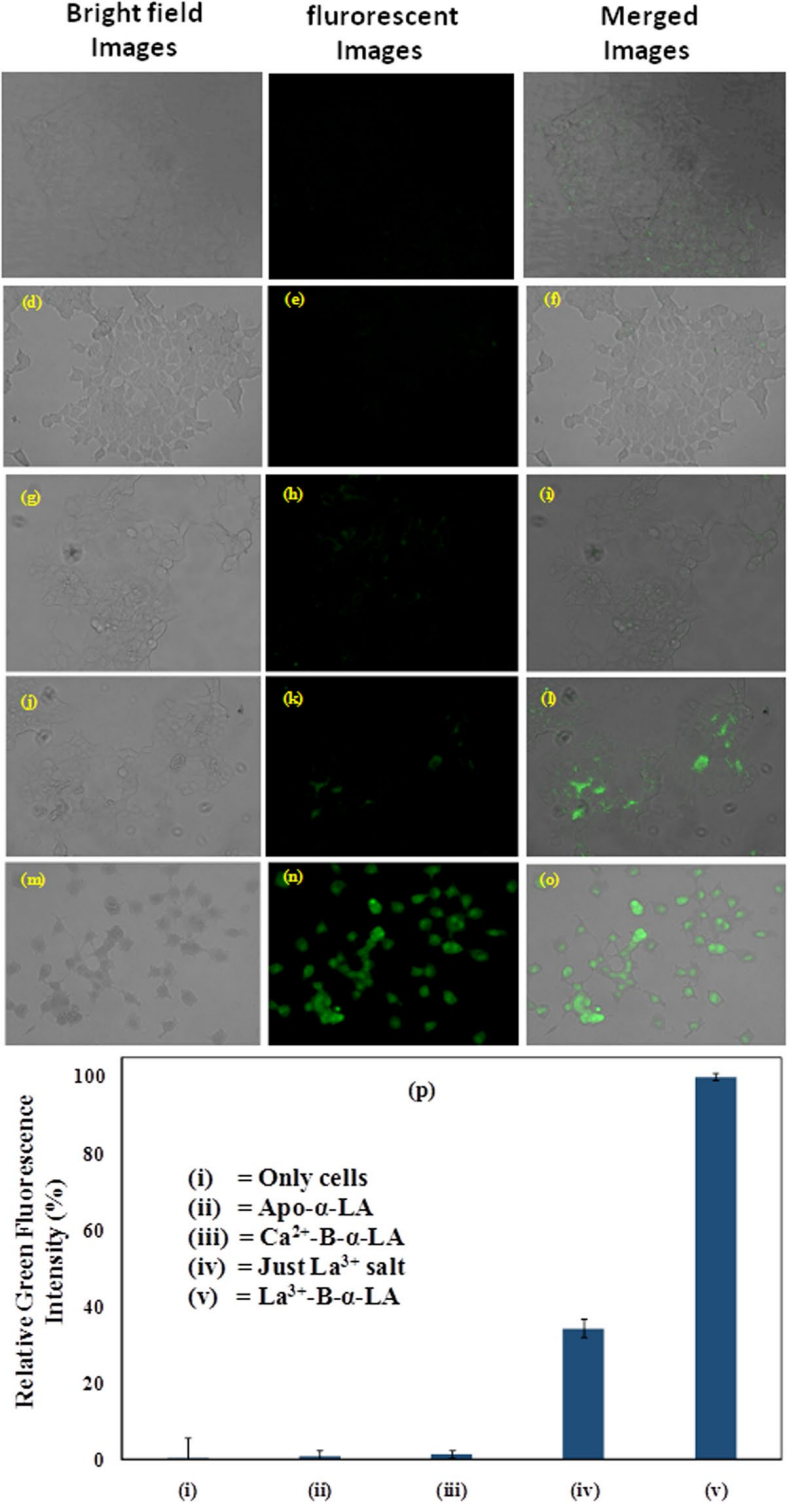
Fluorescence microscopy images of MCF-7 cells: (**a**–**c**) Are untreated. (**d**–**f**) Are for apo-B-α-LA. (**g**–**i**) Are for Ca^2+^-B-α-LA. (**j**–**l**) Are for just La^3+^ salt. (**m**–**o**) Are for La^3+^-B-α-LA. (**p**) Relative green fluorescence intensity for the studies carried out with MCF-7 cells when treated with that mentioned in the figure.

The apoptotic cell death was demonstrated in MCF-7 cells by fluorescence microscopy in the presence of La^3+^-B-α-LA complex, by carrying out the experiments using caspase 3/7 dye^31^. The microscopy studies carried out in this regard include, (i) untreated cells, and cells treated with (ii) 15 µM apo-B-α-LA, (iii) 15 µM Ca^2+^-B-α-LA, (iv) 200 µM of La^3+^ salt and (v) 15 µM La^3+^-B-α-LA complex. In case of (i), (ii) and (iii), no green fluorescence was observed suggesting that there is no role in activating caspase 3/7 driven apoptosis. However, La^3+^ treated cells, i.e., (iv), exhibited weak green fluorescence indicating that La^3+^ treatment alone can activate caspase 3/7 mediated apoptosis to some extent. Though the cells adopted round shape due to the treatment, these have not undergone apoptosis. In case of the cells treated with La^3+^-B-α-LA complex, i.e., (v), almost all the cells exhibited typical green fluorescence as well as distorted cell morphology (Fig. 8) indicating the activation of caspase 3/7 pathway which is characteristic of apoptotic cell death^32^. The integrated green fluorescence intensity is >3 times higher when the MCF-7 cells were treated with La^3+^-B-α-LA complex as compared to the same when treated with simple La^3+^ salt (Fig. 8p).

## Concluding Remarks

Both the absorption and emission spectra clearly showed the interaction of La^3+^ with apo-B-α-LA, and ICP-AES and the ITC showed 1:1 protein: La^3+^ complex. The crystal structure of La^3+^-B-α-LA complex was determined at a resolution of 1.85 Å. To our knowledge, this is the first La^3+^ complex of B-α-LA. The structure revealed the presence of La^3+^ occupying the Ca^2+^ binding site of native B-α-LA but with some primary coordination sphere changes as compared to that of the Ca^2+^- and or apo- protein. Thus, the La^3+^ exhibit eight coordination with dodecahedral geometry, while the Ca^2+^ was in distorted pentagonal bipyramidal geometry with seven coordination. As the La^3+^ ion occupies the Ca^2+^ cleft in the protein, this is not expected to alter the spectral features to any great extent. The CD spectra indeed showed that the loss of α-helical structure is minimum, which can also be gauzed from the crystal structure. The average B-factors of the main chain atoms are 29.0, 38.3 and 48.4 Å^2^ respectively for La^3+^-B-α-LA, apo-B-α-LA and Ca^2+^-B-α-LA structures supporting that La^3+^ complex of protein is more rigid than the apo- as well as holo (Ca^2+^)- proteins. The La^3+^-B-α-LA complex at a concentration of 0.5 mg/ml specifically kills the breast cancer cells (MCF-7 cells) to an extent of >90% sparing the healthy ones, and hence this protein – La^3+^ complex is specific to the cancer type. The differential cell proliferation results obtained among these three cancer cells seems to be dependent on the nature of the receptor sites present on the corresponding cells, though the details of the same deserves a specialized study. Recently, we found greater anticancer activity in case of MCF-7 cells over the other cancer cells even in case of α-LA coated on AuNPs where the conformation of the protein would be different from the apo- or holo- protein, supporting that the structural change in the protein has role in the anticancer activity^20^.

The cell death has been confirmed to arise from apoptosis based on the studies carried out in presence of caspase 3/7 dye by fluorescence microscopy. The anticancer activity exhibited by the La^3+^ complex is much greater as compared to the literature reported BAMLET, which is only ~70%. While the oleic acid stabilizes the partial unfolded structure of the protein to result in BAMLET, in the present case the La^3+^ induces structural changes which are more prone for anticancer activity. Our extended fluorescence microscopy studies reveal that the green fluorescence intensity observed for La^3+^-B-α-LA complex is greater by 3 fold as compared to the same when these were treated with simple La^3+^ salt. In fact, the average B-factor and r.m.s.d. supports this when the corresponding parameters of the La^3+^-B-α-LA complex are compared with apo-/holo(Ca^2+^)-protein and or the lanthanide ion alone. Comparison of the CD spectra of La^3+^-B-α-LA complex with that of the literature reported BAMLET^33^ supported that the La^3+^ induces a different structure and this supports the greater anticancer activity exhibited by the La^3+^-B-α-LA complex as compared to the BAMLET in MCF-7 cells.

## Methods

Apo-B-α-LA was procured from Sigma Aldrich Chem. Co., and used in all the experiments without further purification. The lanthanum perchlorate salt was prepared starting from lanthanum oxides followed by treating the reaction mixture with perchloric acid and recrystallizing the product. A 10 mM Tris-HCl buffer at pH 7.4 was used for all the experiments unless otherwise mentioned.

Both the healthy and the cancer cells used in the present study were obtained from National Centre for Cell Science, Pune, India. Dulbeco’s Modified Eagle Medium (DMEM) and DMEM without phenol red, Dulbecco’s Phosphate Buffered Saline (DPBS), Fetal Bovine Serum (FBS) and coverslips for fluorescence microscopy were purchased from Sigma-Aldrich, USA. Caspase 3/7 green detection reagent was procured from Thermo Fischer scientific.

### Spectroscopy

UV-Visible absorption studies were performed on Varian instrument. One mL of 0.015 mM (25 µl of 1 mg/mL) apo-B-α-LA was taken into a cuvette and titrated against 5 mM La^3+^ such that the metal ion to protein mole ratio varied from 0 to 60 folds (by adding incremental addition of 0.5 µl metal ion solution at each time) and subtracted from the background at a scan rate 200 nm/min. Fluorescence spectral studies were performed on a Varian instrument at the same concentrations and mole ratios. The experiments were performed in quartz cuvette of path length 1 cm and scan speed of 200 nm/min. Fluorescence spectra were measured by exciting the solutions at 280 nm and 295 nm. Other details are same as that used for the absorption spectra. Far–UV CD spectra were recorded on JASCO-810 using Quartz cuvette of 0.1 cm path length. The CD spectra were accumulated at room temperature at a scan speed of 100 nm/min between 190–270 nm. Each time, 0.5 mL of 35 µM apo-B-α-LA was taken for CD measurements. For inductively coupled plasma atomic emission spectroscopy (ICPAES), a 1 mg/mL of apo-B-α-LA and La^3+^ (at 1:10 ratio) were taken and incubated overnight and then the samples were diluted to 10 mL and were dialysed in 10 mM Tris-HCl buffer at pH 7.4 for 6 hours. The samples were analysed with ICPAES before and after the dialysis, and the metal concentration was evaluated.

### Isothermal titration calorimetry (ITC)

The calorimetric titrations were performed at 25 °C with a microcal isothermal titration calorimeter from GE Healthcare (Northampton, MA, USA). The concentration of the protein used was 70 µM and this was titrated against 20 µl of 5 mM of La^3+^salt solution. The buffer was similarly titrated with same metal ion concentration and this was subtracted to give the final thermogram. All the solutions were degassed for 30 minutes prior to the start of the experiment. A good fit to these data was done using the Origin software 8.0 version.

### Crystallization

The protein, apo-B-α-LA was dissolved in metal ion free water. The final concentration of the protein used for crystallization was 25 mg/mL. Apo-B-α-LA with La^3+^ ion was crystallized using hanging drop vapour diffusion method at 295 K. The crystallization drops were set up by mixing 2 µl of protein solution with 2 µl of mother liquor and 0.5 µl of 2 mM lanthanum perchlorate. The crystallization drops were equilibrated against 300 µl of mother liquor. Optimization of protein to La^3+^ ion concentration ratio was performed prior to crystallization set up to avoid precipitation in the crystallization drop. The best crystals were obtained using precipitant containing 0.1 M Tris-HCl at pH 6.0 and 2 M ammonium sulphate. The crystals appeared within one week of crystallization set up and these grew to their maximum size of 0.3 × 0.25 × 0.15 mm in three weeks.

### Diffraction data collection and data processing

The crystals were cryo-protected using the reservoir solution also containing 30% (v/v) of glycerol. A single crystal was picked up from the crystallization drop using cryo-loop and quickly transferred to the cryo-protectant solution. Immediately after that, the crystal was flash cooled by transferring it to the liquid nitrogen stream at 100 K. The diffraction data set was collected by rotation method with 0.5° oscillation per image. The data set was collected at the Protein Crystallography Facility of Indian Institute of Technology Bombay using Cu Kα X-ray source generated by a Rigaku Micro Max-007 HF diffractometer fitted with R-Axis IV++ image plate detector. The indexing, integration and scaling of the data set were performed by XDS software package^34^. The intensities were converted to structure factors with program modules F2MTZ and CAD of CCP4^35^. The data collection statistics is presented in Table 1.

### Structure determination and refinement

The structure of La^3+^bound apo-B-α-LA was determined by molecular replacement method using the molecular replacement module of the PHASER program^36^. The A-chain of Ca^2+^-B-α-LA crystal structure (PDB ID = 1F6S)^27^ was used as a search model for the initial phase determination. The calculation of Matthews’ coefficient^37^ showed the presence of one molecule in the asymmetric unit with *V*_M_ value of 2.06 A^3^ Da^−1^ which corresponds to 40% solvent content. After finding correct orientation of the protein molecule by PHASER, initial few cycles of refinement of the model was done by REFMAC^38^. The analysis of the initial sigma weighted *F*_*o*_-*F*_*c*_ electron density map showed the presence of lanthanum ion in the metal binding cleft of B-α-LA. After placing the La^3+^ ion inside the protein molecule, repeated cycles of refinement were done using REFMAC and manual model building was performed using COOT^39^. Water and glycerol molecules, and sulphate ions were progressively added at peaks of the sigma-A weighted *F*_*o*_-*F*_*c*_ electron density map higher than 3σ level while monitoring the decrease of *R*_free_ and improvement of the overall stereochemistry of the protein structure. The last two residues could not be built because of the lack of features of these residues in the electron density map. The statistics of the structure refinement is presented in Table 1. The structural figures were generated using PyMOL version 1.3^40^. The secondary structural elements were assigned using Dictionary of Secondary Structure of Proteins (DSSP) server^41^.

### Cell viability study

Sulforhodamine B (SRB) assay was performed to evaluate cell viability with L-929, N1H3, HeLa, KB and MCF-7 cell lines. The cells were seeded into 96-well plates at densities of 1 × 10^4^ cells per well and incubated for 24 h. Different concentrations of the samples were added to the cells and incubated for 24 h at 37 °C in the atmosphere of 5% CO_2_. Thereafter, the cells were washed thrice with phosphate buffer saline (PBS) and processed for SRB assay to determine the cell viability. For this, the cells were fixed with a solution of 50% trichloroacetic acid and stained with 0.4% SRB dissolved in 1% acetic acid. Cell-bound dye was extracted with 10 mM Tris buffer solution at pH 10.5 and then the absorbance was measured at 560 nm using a plate reader. The cell viability was calculated as the ratio of the absorbance of the sample to the control, and was expressed in %.

### Apoptosis study using caspase 3/7 dye

MCF-7 cells were seeded on cover slips in a 6 well plate at a density of 10^4^ cells/mL in DMEM containing 10% FBS and a 0.1% antibiotic solution and incubated for 24 h at 37 °C and 5% CO_2_ for adherence. Once MCF-7 cells become fully confluent, these were treated with 15 µM of La^3+^-B-α-LA prepared in DMEM without phenol red (treatment media) and further incubated for 24 h. After incubation, the medium was removed from each well and the cells were washed carefully with PBS. 10 μL of ready to use caspase 3/7 dye prepared in 1 mL of PBS was added to the cells. The cells were further incubated at 37 °C for 15 min. After incubation, the cover-slip was removed from each well and mounted on glass slide using glycerol as mounting medium. Imaging was carried out using FLoid® Cell Imaging Station (Life Technologies) using 20X objective.

## Supplementary information

Supporting Information
