# Supplementary material for: Cytotoxicity of apo bovine α-lactalbumin complexed with La3+ on cancer cells supported by its high resolution crystal structure
Source: Sci Rep. 2019 Feb 11;9:1780. doi: 10.1038/s41598-018-38024-1 (PMC6370903; doi:10.1038/s41598-018-38024-1)
Supplement: Supplementary file 1 — Supporting Information [file 41598_2018_38024_MOESM1_ESM.docx]

**Cytotoxicity of apo bovine α-lactalbumin complexed with La^3+^ on cancer cells supported by its high resolution crystal structure**

Deepthi S. Yarramala^1^, Prem Prakash^2^, Dnyanesh S. Ranade^3^, Sejal Doshi^4^, Prasad P. Kulkarni^3^, Prasenjit Bhaumik^2,^ and Chebrolu Pulla Rao^1*^

^1^Department of Chemistry, Indian Institute of Technology Bombay, Powai, Mumbai – 400 076, India.

^2^Department of Biosciences and Bioengineering, Indian Institute of Technology Bombay, Powai, Mumbai – 400 076, India.

^3^Agharkar Research Institute, Pune – 411 004, India.

^4^Department of Metallurgical Engineering and Materials Science, Indian Institute of Technology Bombay, Powai, Mumbai – 400 076, India.

* Corresponding author, E-mail for correspondence: cprao@iitb.ac.in

**Supplementary Materials**

**Contents**

**S01.** ICPAES data 02

**S02.** The bond angles observed in the primary coordination sphere of La^3+^-B-α-LA 02

**S01.** ICPAES data.

**Table S1**: ICP-AES analysis data.

|  | **Ca^2+^**  **(ppm)** | **Zn^2+^**  **(ppm)** | **Ln^3+^**  **(ppm)** |
| --- | --- | --- | --- |
| Apo B-α-LA | 0.05 | 0.004 | ND |
| Apo B-α-LA +La^3+^ | 0.16 | 0.02 | 0.94±0.02 |

**S02.** The bond angles (°) observed in the primary coordination sphere of La^3+^-B-α-LA:

O1-La^3+^-O2: 74.2, O1-La^3+^-O3: 135.6, O1-La^3+^-O4: 110.8, O1-La^3+^-O5: 88.6, O1-La^3+^-O6: 129.4, O1-La^3+^-O7: 68.1, O1-La^3+^-O8: 76.9, O2-La^3+^-O3: 121.3, O2-La^3+^-O4: 84.8, O2-La^3+^-O5: 92.1, O2-La^3+^-O6: 58.7, O2-La^3+^-O7: 115.2, O2-La^3+^-O8: 150.5, O3-La^3+^-O4: 107.0, O3-La^3+^-O5: 56.9, O3-La^3+^-O6: 65.6, O3-La^3+^-O7: 121.6, O3-La^3+^-O8: 79.2, O4-La^3+^-O5: 158.6, O4-La^3+^-O6: 83.9, O4-La^3+^-O7: 63.5, O4-La^3+^-O8: 110.8, O5-La^3+^-O6: 76.5, O5-La^3+^-O7: 135.5, O5-La^3+^-O8: 81.7, O6-La^3+^-O7: 144.5, O6-La^3+^-O8: 147.4 & O7-La^3+^-O8: 56.9.
